# A Mobile Ecological Momentary Intervention for Reducing Experiential Avoidance in the Context of Rumination: Protocol for a Randomized Controlled Trial

**DOI:** 10.2196/66067

**Published:** 2025-05-27

**Authors:** Steven Barnes, Marta Szastok, Małgorzata Para, Fabian Morawiec, Maciej Grzeszczuk, Szymon Wójcik, Barbara Karpowicz, Pavlo Zinevych, Anna Jaskulska, Wiesław Kopeć, Monika Kornacka

**Affiliations:** 1 Emotion Cognition Lab SWPS University Katowice Poland; 2 XR Centre Polish Japanese Academy of Information Technology Warsaw Poland; 3 Kōbō Association Warsaw Poland

**Keywords:** rumination, experiential avoidance, mHealth, ecological momentary intervention, transdiagnostic processes, low-intensity intervention, repetitive-negative thinking, cognitive avoidance, psychoeducation

## Abstract

**Background:**

Rumination is a transdiagnostic process present in several psychological disorders, involving repetitive negative thinking that individuals may perceive as difficult to control. While the roles of numerous mechanisms underlying rumination have been supported, experiential avoidance (EA) still lacks empirical backing, despite a strong theoretical foundation, partly due to difficulties in examining EA in an ecologically valid context. One promising approach to addressing this challenge is through reducing EA using mobile health (mHealth) and ecological momentary intervention (EMI), and assessing any subsequent decrease in rumination’s deleterious outcomes.

**Objective:**

This paper outlines the protocol for a randomized controlled trial using a novel mHealth EMI to address EA in the context of rumination. The app was developed by a multidisciplinary team, incorporating feedback from potential end users.

**Methods:**

Consenting individuals (target N=60) who meet the inclusion criteria (self-reporting problems with repetitive negative thinking) will be randomly assigned to 1 of 4 conditions: (1) intervention with therapist support and daily sampling, (2) intervention without support and with daily sampling, (3) partial intervention (emotion validation EMI only) with daily sampling, or (4) control (daily sampling only). The intervention consists of a series of modules delivered over 4 weeks, with assessments conducted before and after intervention, and again at 1-month follow-up (plus an additional 3-month follow-up for intervention participants). Data will be collected both through online self-report assessments and via the app itself. The potential of the EMI to modify the maladaptive feature of repetitive negative thinking will be assessed using mixed-design ANOVA, while the links between avoidance-mood and rumination-mood, in terms of the moderating effect of trial condition, will be evaluated using multilevel models. These will be assessed as primary outcomes. Secondary outcomes are the effects of a supporting therapist on postintervention outcomes in the intervention groups, and time spent using the app as a measure of engagement (analyzed using the mixed-design analysis of covariance). Compliance will be defined as completing both of the first 2 weeks of intervention content in full, and 5 out of the 6 exercises from weeks 3 and 4. Additionally, the study will control for both the amount of time spent in the app and the length of responses to open-ended questions.

**Results:**

Recruitment and enrollment for the trial are expected to begin in May 2025 and be completed by July 2025. Data collection will conclude once the target sample size for each of the 4 conditions has been reached. The main results of the trial are expected to be submitted for publication in October 2025.

**Conclusions:**

The outcomes of this research trial will not only provide insights into the clinical capabilities of the app, including its usability and acceptability in real-world contexts (and its potential future viability as a scalable product), but will also offer valuable theoretical insights into the role of EA in maladaptive rumination.

**Trial Registration:**

ClinicalTrials.gov NCT06570694; https://clinicaltrials.gov/ct2/show/NCT06570694

**International Registered Report Identifier (IRRID):**

PRR1-10.2196/66067

## Introduction

### Rumination and Psychopathological Outcomes

Epidemiological data suggest that comorbidity is the rule rather than the exception [1], with it frequently observed in at least half of the diagnosed population [2]. The experience of comorbidity has significant negative implications for clinical severity and prognosis [3,4]. Additionally, the persistently moderate outcomes of treatment programs and high levels of posttreatment relapse indicate a still limited understanding of the mechanisms that initiate, underpin, and maintain a number of psychiatric disorders [5].

Transdiagnostic models represent a shift away from disorder-centric approaches to the understanding and treatment of psychological disorders. Instead of focusing on a specific nosological diagnosis, they suggest focusing on processes that may serve as risk, maintenance, and relapse factors across multiple psychological disorders, thereby more effectively addressing the frequent comorbidity observed in mental disorders. Interventions based on transdiagnostic approaches have demonstrated positive outcomes in reducing symptom presentation across several disorders [3,6-10].

Rumination is a transdiagnostic process present in several psychological disorders [11]. Traditionally, rumination was explored in the context of mood disorders and defined as a mode of responding to distress that involves repetitively and passively focusing on symptoms of distress and the possible causes and consequences of these symptoms. Nevertheless, rumination is now recognized as being involved in numerous psychological disorders and adopts a broader definition—a form of repetitive thinking about 1 or more concerns perceived to be difficult to control [12]. While rumination may not necessarily be wholly maladaptive, it can become so when overgeneralized and used inflexibly [13], in which case it has been shown to impair emotional regulation and increase negative affect [14]. Thus, one can expect that addressing this maladaptive transdiagnostic process will result in a reduction of psychopathology symptoms (eg, [11]). Experimental studies that induce rumination in distressing contexts demonstrate elevated depressed and anxious mood [15]. Similarly, self-report studies find rumination to be a risk factor in numerous issues, including as a risk factor and consequence of depressive symptoms [16]; the onset, number, and duration of major depressive episodes [17-19]; and the development of generalized anxiety [19], social anxiety [19], eating disorders [19], posttraumatic stress [20], and borderline symptoms [21]. In addition to its symptomatological impact, rumination has also been shown to reduce the efficacy of psychological interventions [12].

Currently, several models explain the maladaptive nature of rumination—these involve abstract processing and dysregulation at the goal/action identification level (ie, adapting a specific processing mode to current requirements [22]); impairments in executive or attentional functioning [23,24]; or rumination as a learned, habitual response [13,23,24]. A recent H_Ex_A_Go_N model of rumination attempts to integrate the most prevalent theories with a relatively strong empirical background on the mechanisms maintaining maladaptive rumination, identifying rumination as a *h*abit, *ex*ecutive function impairment, *a*bstract processing mode, *go*al discrepancies triggering rumination, and the *n*egative valence of rumination content [12]. Thus, the authors suggest that rumination becomes a maladaptive, transdiagnostic process when it is used as a habitual, overgeneralized response to negative affect [12,25].

Nevertheless, the literature suggests that another mechanism could be involved in making rumination maladaptive and in reinforcing it as a habitual response—namely, experiential avoidance (EA). Although this mechanism features prominently in the theory of repetitive negative thinking (RNT), it has received very limited empirical support. This is not necessarily due to a lack of supportive data (eg, [25]), but rather to the small number of studies testing this hypothesis, largely because of the challenges involved in evaluating EA in empirical research.

### Mechanisms Underpinning Rumination: Examining the Role of Experiential Avoidance

EA refers to attempts to eschew emotional distress by engaging in maladaptive behaviors that temporarily allow escape from negative emotional experiences. Through the use of EA, individuals may disconnect from unpleasant emotions by altering the form or frequency of these experiences [26], even when such avoidance causes behavioral harm by interfering with goal pursuit and impairing learning through experience. This often leads to the adoption of additional maladaptive coping strategies, such as substance use [27].

Engagement in EA has adverse effects on the development, maintenance, and modification of chronic psychological disorders such as depression, due to its tendency to generate negative mood and initiate a maladaptive cycle of emotional distress and avoidance [28]. Consequently, EA may represent a core vulnerability for emotional distress [29]. To this end, randomized controlled trials delivering therapeutic interventions focused on reducing EA (such as acceptance and commitment therapy) have found that decreases in EA are associated with reductions in depressive and anxious symptoms [28,30,31].

As a mechanism underpinning rumination, however, despite some empirical evidence emerging to examine its role [12,32], EA continues to lag in terms of supporting data. This is despite the theoretical basis for the role of EA in rumination [22,33], where rumination serves as a means to avoid increased arousal and distress through an overgeneral, abstract style of thinking, despite its maladaptive implications for real-world problem-solving and long-term deleterious consequences for emotion regulation [22,24,25]. Of note, a study examining multiple indices of avoidance reported differential outcomes across the 3 modes of investigation (self-report, dichotic listening tasks, and psychophysiological measures), lending only partial support to the avoidance conceptualization of rumination [34].

Difficulties in examining the role of EA may, in part, be due to several methodological challenges associated with assessing EA in ecologically valid contexts. While experimental studies appear to lend support to an EA conceptualization of rumination [29,35], the tasks used to evaluate it in laboratory settings (eg, approach-avoidance task or dichotic listening task) do not reflect the real-world occurrences of EA. Similarly, while self-report evaluations connect EA with rumination [34,36], such data rely heavily on retrospective recollection, assuming that participants were aware of their use of (and the extent of their use of) avoidance. One alternative approach to examining the role of EA may involve adopting the experimental method, that is, reducing EA through ecological momentary intervention (EMI) [37], and examining any resulting reduction in the deleterious outcomes of rumination.

### Mobile Health as a Vehicle for Ecological Momentary Intervention

The availability of mental health services has struggled to keep pace with the growing demand for treatment [38,39]. One means of addressing this imbalance has been through the use of digital interventions, which offer the potential to provide accessible, rapid, and scalable pathways to intervention [40].

Mobile health (mHealth) apps represent a dimension of digital health provision and allow for the delivery of validated psychological interventions on-demand via smartphones. Because of the ubiquity of smartphones [41] and the opportunities afforded for personalization of provision, mHealth apps offer the potential to deliver rapidly scalable interventions that can expand the reach of mental health services while overcoming a number of barriers faced by traditional forms of treatment [42].

EMI further represents an opportunity to utilize ubiquitous devices, such as smartphones, to integrate treatment protocols into people’s everyday lives. Treatment can be delivered in natural environments as either a standalone intervention or as an addendum to existing treatment [43], and can be based on the outcomes of real-time daily sampling assessments also conducted in the real world (ecological momentary assessment [EMA]). The use of real-time assessment data allows for provision to be personalized and tailored to the general requirements of an individual client and, on a micro-level, as a form of opportune intervention within a specific environmental context [44]. As an assessment tool, EMA also helps overcome the limitations of global self-report measures, where retrospective recollections of emotions and events may be distorted by subsequent use of regulation strategies [45].

Literature has begun to explore the capabilities of mHealth EMIs as tools for mental health intervention, with promising results emerging across a range of conditions [46,47]. In addition to their potential therapeutic benefits, EMIs represent several methodological opportunities to improve ecological validity. First, the use of ubiquitous devices such as smartphones allows research to be conducted in real-world settings, reducing the reliance on laboratory-based experimental studies, where outcomes may, in part, be a product of the artificiality of the environment. Instead, the real-world nature of treatment delivery allows for the examination of intervention effects (eg, in the context of transdiagnostic processes) in real-world contexts. Second, while EMAs may not always be conducted in “real time” (eg, at the moment of symptom experience), data can be collected within a short period thereafter. The implementation of regular EMA also reduces the reliance of treatment efficacy studies on postintervention self-report data, which can be vulnerable to retrospective bias, particularly for individuals experiencing depressive disorders [48,49].

Uptake and engagement with mHealth, however, may be negatively associated with poor app design, technical issues, digital literacy, poor suitability of content, lack of professional support, and pathological factors (eg, low motivation), when content is delivered in a manner unsuited to user requirements, or when engagement is considered by users to be too labor intensive [50-55]. Numerous factors at both the individual and group levels have been identified that may affect the intention to use or uptake of digital interventions [56,57]. Effective multidisciplinary collaboration in app development, which acknowledges the intricate needs of target end users and incorporates these into the design of the intervention, is essential for improving acceptability, usability, and accessibility [58], particularly when interventions are deployed in unsupervised settings [59]. EMIs require unique considerations in their design, deployment, and evaluation [60], which help specify the procedures for collecting and examining user data and the circumstances under which the intervention may be delivered. Where EMIs actively respond to user data in real time, processes for determining appropriate intervention (while respecting user privacy) must also be considered.

### Study Objectives

The scientific verification of processes that underpin several transdiagnostic maladaptive cognitive processes and psychopathological outcomes has several benefits. First, successfully identifying such processes advances the theoretical understanding of transdiagnostic processes themselves and their contributing roles in psychopathology. Second, it has the potential to improve several psychological treatment programs by identifying valuable targets for intervention [21], which is particularly valuable in treating cases of comorbidity. Using EMI and EMA in clinical trials offers a unique opportunity to evaluate the mechanisms underlying transdiagnostic processes that are difficult to assess through traditional self-report or laboratory methods (eg, avoidance). This study aims to examine whether the avoidance-focused EMI for rumination can modify the maladaptive feature of RNT, as understood through the link between daily RNT and well-being, depressive, and anxiety symptoms, compared with an active control group. We anticipate that the link between daily rumination and its maladaptive outcomes (lower well-being and higher depressive and anxiety symptoms) will be weaker in the intervention groups compared with the control group. Additionally, the study aims to confirm the role of avoidance in RNT.

Furthermore, by splitting the intervention condition into 2 groups (1 receiving concurrent therapist support and 1 not receiving concurrent therapist support), the study aims to evaluate whether intervention outcomes may be enhanced by the added availability of a supporting therapist [61].

Finally, the study aims to examine whether the impact of the avoidance-focused EMI for rumination on depressive and anxious symptoms can, compared with an active control group, be mediated by changes in beliefs about emotion (valuation of negative emotions) [62]. Similarly, the study aims to assess the EMI in terms of similar mediation effects of beliefs about rumination [63].

## Methods

### Study Design

**Figure 1:**
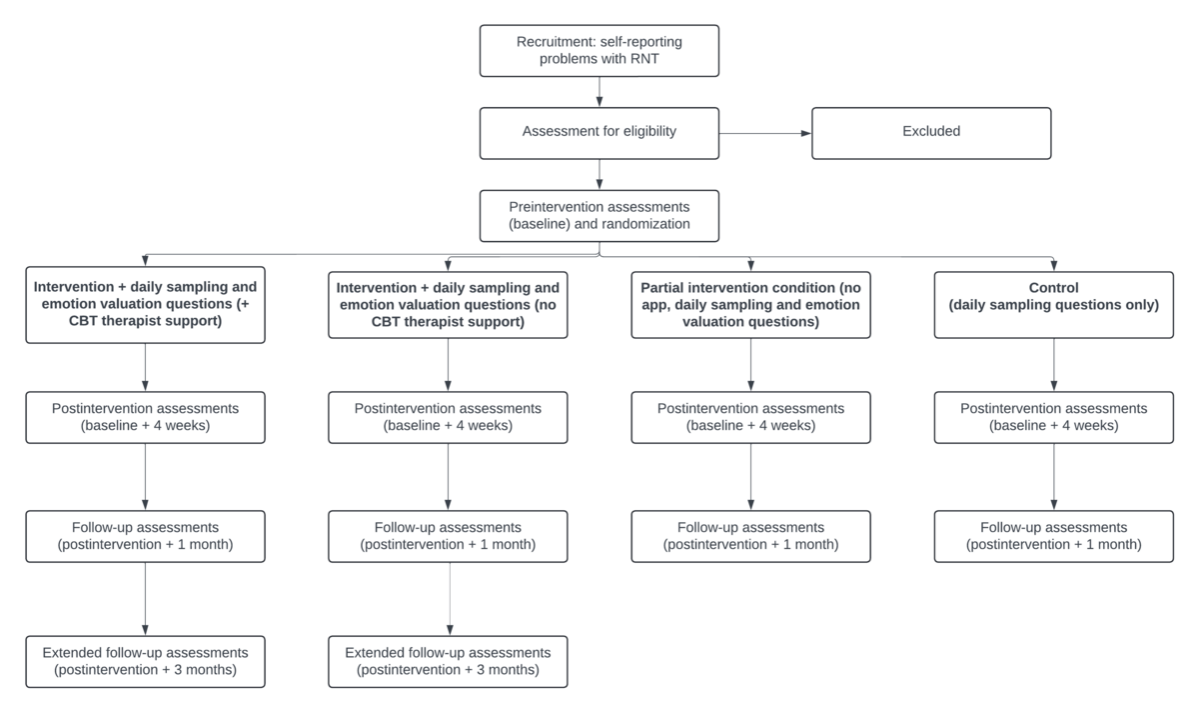
Graphical representation of the randomized controlled trial protocol. CBT: cognitive behavioral therapy; RNT: repetitive negative thinking.

The study takes the form of a 4-arm, parallel-group randomized controlled trial. Total study participation will last approximately 2 months, including preintervention eligibility screening, a 4-week intervention period using the app content and daily sampling, postintervention assessments at the end of the intervention, and a 1-month follow-up period (during which the app will remain available for self-directed use without daily sampling), concluding with follow-up assessments and measurement of any engagement with the app during this period. At this point, control participants will receive the app (minus daily sampling) and guidance on how to use the content. An initial follow-up period of 1 month has been chosen for ethical purposes to minimize the amount of time control participants will wait before receiving the app. For participants in the intervention groups, the study aims to conduct an additional evaluation of outcomes 3 months following the intervention. A graphical summary of the study design is presented in Figure 1.

Participants will be randomly assigned, using a random-number generator, to 1 of 4 conditions: (1) intervention condition (therapist support); (2) intervention condition (no therapist support); (3) partial intervention (daily sampling and emotion valuation questions); or (4) control group (only daily sampling questions). Active control groups will be utilized to avoid artificially inflating comparisons with intervention condition outcomes [64]. The specific group allocations and the nature of other study conditions will be concealed from participants. Block/random stratification will not be used, as participants will be randomly allocated to 1 of the groups after agreeing to participate in the study. During analysis, demographic characteristics across groups will be compared, and any significant differences identified will be included as control variables.

Therapist support has been shown to improve outcomes in studies of digital mental health interventions [61]. In this study, therapist support will take the form of asynchronous written communication with a cognitive behavioral therapy (CBT) practitioner—either fully qualified or in at least their second year of training—via WhatsApp (Meta Platforms, Inc). This support will be available 2 times a week to relevant participants at specific times predetermined by the therapist. Participants will have the option to utilize this support, but will not be directed to do so. Supporting therapists will be paid at their standard hourly rate for regular patients and will receive an introduction to the app and intervention—while remaining blinded to the full aims of the study—through an online workshop conducted by members of the research team experienced in delivering rumination-focused cognitive behavioral therapy (RFCBT), before the study.

### Participants and Power Analysis

Participants who self-report problems with RNT will be sought. The first participation in the trial is planned to commence in May 2025. As the study will be conducted remotely and recruitment will continue beyond this date, participants are expected to begin their engagement asynchronously.

To be eligible for the trial, participants must self-report problems with RNT. Participants will be excluded if (1) they are under 18 years of age at the start of the trial; (2) they are currently receiving any form of psychotherapy; or (3) they are currently receiving any form of psychiatric medication. Participants with a psychiatric diagnosis will not be excluded, provided they meet the inclusion criterion and do not meet any of the exclusion criteria listed above.

Sample size was calculated using G*Power version 3.1 [65] for a repeated-measures design with 4 independent conditions measured at 3 time points and assuming a medium effect size. This indicated a required sample size of 60 with equal randomization of participants across groups, which aligns with previous intervention studies in the field [66-68]. As our initial feasibility study during the development of the app content experienced an attrition rate of approximately 60% [69], this study aims to recruit at least double that number to mitigate the risk of significant dropout throughout the trial.

### Recruitment Strategy

Participants will be recruited through online advertising on social media platforms (Facebook/Instagram; Meta Platforms, Inc) and via the laboratory website, where a contact email address will be provided for interested individuals to express their interest in participating. Full study information will be sent by email to all interested participants, and informed consent will be obtained via email response before the delivery of the app and the commencement of participation. To minimize information sharing across groups, participants will not have access to one another’s contact or identity details. Additionally, the current version of the app does not allow users to contact other participants or to determine how many others are using the app.

Recruitment is expected to begin in early May 2025 and will continue until the target number of participants in each condition has been reached. Participants may withdraw from the study at any time by contacting the research team via a designated email address provided in the study information form or through a link within the app. All data associated with participants who choose to withdraw will be fully removed from the study and destroyed. Participants will receive financial compensation of €50 (US $56) for taking part in the study.

### Measures

#### Pre-Post and Follow-Up Measures

The following measures will be collected from all participants before/during the intervention, after the intervention, and at follow-up:

The Perseverative Thinking Questionnaire (PTQ) [70], a 15-item self-report measure that assesses the main characteristics of RNT: (1) core features (9 items); (2) unproductiveness (3 items); and (3) capturing mental capacity (3 items). Responses are recorded on a scale from 0 (never) to 4 (almost always). The PTQ has demonstrated good internal consistency and test-retest reliability [70], not only in clinical samples, but also in nonclinical populations and when administered online.

Emotions Beliefs Questionnaire (EBQ), a 16-item self-report questionnaire designed to measure the controllability of emotions and respondents’ beliefs about the usefulness of emotions, which also demonstrates good internal consistency [71]. Respondents answer using a 7-point Likert scale, ranging from “1” (totally disagree) to “7” (totally agree), with higher scores indicating a belief that emotions are uncontrollable or useless.

The Positive and Negative Beliefs About Rumination Scales (PBRS and NBRS, respectively), which comprise 2 scales (positive beliefs: 9 items; negative beliefs: 13 items) that measure metacognitive beliefs related to depressive rumination. Respondents rate the extent to which they agree with each item using a 4-point Likert-type scale, ranging from “1” (do not agree) to “4” (agree very much), with higher scores on each subscale indicating greater positive or negative beliefs, respectively. Both the PBRS and NBRS demonstrate adequate to good internal consistency [72].

Hospital Anxiety and Depression Scale (HADS), a measure of the possible and probable presence of anxiety disorders and depression, comprising 7 items, developed by Zigmond and Snaith [73]. It has been shown to possess good internal consistency, with sensitivity and specificity comparable to the General Health Questionnaire [74]. Respondents rate each item on a scale of 0-3, with higher total scores for each subdomain (anxiety and depression) indicating a greater presence of the disorder. Thresholds for determining the clinical presence of the disorder are as follows: 0-7=normal, 8-10=borderline abnormal (borderline case), and 11-21=abnormal (case).

Cognitive Avoidance Questionnaire (CAQ), developed by Gosselin et al [75], is a 25-item measure of 5 worry-related cognitive avoidance strategies (thought suppression, thought substitution, distraction, avoidance of threatening stimuli, and transformation of images into thoughts), demonstrating good to excellent internal consistency and test-retest reliability [76]. The scale comprises 5 subscales, each containing 5 items that represent individual cognitive avoidance strategies (avoidance of threatening stimuli, distraction, thought substitution, thought suppression, and transformation of images into thoughts). Respondents answer on a 5-point scale (1=not at all applicable to 5=very applicable), with higher scores indicating a greater presence of cognitive avoidance.

The Brief Experiential Avoidance Questionnaire (BEAQ) is a 15-item short version of the Multidimensional Experiential Avoidance Questionnaire, measuring EA content across 6 dimensions. Respondents provide answers to predetermined statements along a 6-point Likert scale (1=strongly disagree to 6=strongly agree), demonstrating good internal consistency and strong convergence with the original Multidimensional Experiential Avoidance Questionnaire dimensions [77].

Additionally, 2 attentional checks are included in the pre-, post-, and follow-up measures. Participants will be explicitly asked to choose a response option (eg, “This is an attentional check; please select the option ‘sometimes’ in response to this question”).

#### Ecological Momentary Assessment

Users are provided with a daily notification (at a time in the evening of their choosing and for a total of 30 days) to respond to a series of items drawn from existing scales for depression [78], anxiety, well-being, daily goals (and the achievement thereof) [79], emotional experience during the day adapted from Pe et al [80], and RNT [81]. Upon first using the app, users are invited to personalize a time to receive the notification to complete the daily assessment. While a time in the evening is encouraged, flexibility is provided, for example, for individuals working nights. Assessment items can be found in English in Multimedia Appendix 1.

#### User Experience (Contextual, One-Item, Repeated, Timely, Open-Ended [CORTO])

To obtain data regarding the acceptability and usability of the app and to aid its future development, the CORTO (Contextual, One-Item, Repeated, Timely, Open-Ended) method was used, a novel qualitative EMA for obtaining user-experience data in real time [82]. Although originally designed to capture user experiences in relation to games, the method is also relevant in app design as a means of collecting real-time feedback. Data are obtained by presenting users, after each in-app exercise, with 2 closed-ended questions assessing their enjoyment of the app and any difficulties using it, plus 1 open-ended question allowing users to provide detailed, specific feedback on the exercise. Items were amended to replace original instances of “game” with “app” and can be found in English in Multimedia Appendix 2.

#### Additional Control Measures

In addition to the aforesaid measures, data regarding digital health literacy and intervention credibility/expectancy effects will be collected using the eHealth Literacy Scale (eHEALS) and CEQ, respectively [83,84]. The eHEALS assesses respondents’ abilities and skills in sourcing, evaluating, and using electronic health information to address health-related concerns. The scale consists of 8 items measured on a 5-point Likert scale (1=strongly disagree, 7=strongly agree). Total scores range from 8 to 40, with higher scores indicating stronger perceptions of digital literacy in relation to electronic health. The eHEALS-PL demonstrates strong internal consistency (α=.94) [85].

The CEQ consists of 6 items measuring expectations of treatment and the credibility of its rationale in clinical outcome studies. The items are split into 2 sections (“think” and “feel”), with responses for 4 items measured on a Likert scale of 1-9 and for 2 items on an analogue scale from 0% to 100%. Credibility is derived from the first 3 “think” items, and expectancy is derived from the fourth “think” item and the 2 “feel” items. Test-retest reliability is high (expectancy: *r*=0.82; credibility: *r*=0.75; and composite: *r*=0.83) and demonstrates high internal consistency (expectancy: *r*=0.79-0.90; credibility: *r*=0.81-0.86; and composite: *r*=0.84-0.85).

### Intervention

#### OverThinking

OverThinking version 0.3.1(4) is a mobile EMI for iOS and Android platforms, designed to reduce EA in the context of rumination. The app primarily consists of psychoeducation content and related exercises, intended to be delivered over a 4-week period. To examine the effectiveness of the app during the intervention and to assess user experiences with using the app for rumination intervention, several self-report tools are embedded.

#### Development, Content, and User Evaluations

The development of the app was undertaken by a multidisciplinary team of active RFCBT practitioners; research psychologists with experience in transdiagnostic processes, digital mental health interventions, and user experience; and experienced developers of digital tools from the Polish-Japanese Academy of Information Technology.

The intervention comprises psychoeducational content based on RFCBT, an effective intervention for maladaptive rumination [86]. The reduction of EA is targeted through (1) the provision of a functional analysis of rumination to identify the triggers and functions of this process (a classical component of RFCBT), and (2) the modification of beliefs concerning negative emotions and their experience [87,88]. Previous research on the use of RFCBT has also found it to be an effective rumination intervention when delivered in digital contexts [89].

Intervention content was initially developed by members of our team with expertise in RFCBT. The content is divided into 4 weeks, with the first 2 weeks containing several individual modules targeting specific learning outcomes (eg, learning about the function of emotion, learning about the avoidance of emotions). The delivery of informative content is interspersed with several small exercises in different formats (multiple choice or open-ended text entry). Weeks 3 and 4 provide users with a series of exercises designed to consolidate learning and offer an opportunity for users to apply the psychoeducational content to their personal lives. Individual modules are designed to take no longer than 10-15 minutes to complete. The app content is delivered linearly, with subsequent weeks unlocking only once previous ones are fully completed. On each day of the intervention, all users are required to complete an emotion valuation question. All user inputs when completing app content are stored remotely and securely via Firebase (Google LLC), accessible only to the research team and solely for analysis purposes. In addition to user inputs and assessment outcomes, the time taken to complete app content and assessments is also recorded to observe the time burden caused by app use. A summary of the app intervention content can be found in Table 1.

**Table 1:** Overview of intervention content.

Week, module, and module name				Module objectives
**Week 0**				
	**Module 1**			
		Introduction	To provide an introduction to the aims of the app. To introduce the daily assessments and their purpose.	
**Week 1**				
	**Module 1**			
		Introduction	To introduce the aims of module 1 (eg, to inform the user about the nature and function of emotions and their potential negative consequences).	
	**Module 2**			
		The nature of emotions	To examine the nature of depression and anxiety through individual dimensions (thoughts, emotions/feelings, and behavior) in users’ daily lives.	
	**Module 3**			
		The function of emotions	To examine the positive and negative functions of emotions.	
	**Module 4**			
		The avoidance of emotions	To examine the reasons for avoidance of emotions. To identify how users avoid negative emotions. To examine the consequences of avoidance.	
**Week 2**				
	**Module** **1**			
		Introduction	To introduce the second week, which places greater focus on rumination.	
	**Module** **2**			
		Ruminating	To identify and define rumination. Via fictional case studies, to identify situations in which users engage in rumination.	
	**Module 3**			
		Why do we ruminate?	To examine motivations for engaging in rumination. To identify some of the negative consequences of rumination.	
	**Module** **4**			
		Rumination and avoidance	To examine rumination as a means of avoidance/as an emotional regulation strategy.	
**Week 3**				
	**Module 1**			
		Introduction	To introduce the aims of the third and fourth weeks of the intervention—consolidation exercises to apply the psychoeducational content.	
	**Module 2**			
		Exercise 1	Identifying the content and context of the users’ ruminative thinking.	
	**Module 3**			
		Exercise 2	Identifying the triggers of the users’ ruminative thinking.	
	**Module 4**			
		Exercise 3	Identifying the short-term consequences of the users’ engagement in rumination.	
**Week 4**				
	**Module 1**			
		Exercise 1	Examining the use of rumination as a means of avoidance (and identifying what is being avoided).	
	**Module 2**			
		Exercise 2	Reducing/stopping the use of maladaptive rumination. Identifying activities that help users reduce their use of rumination.	
	**Module 3**			
		Exercise 3	Summarizing learning: identifying the context, content, consequences of users’ rumination, and how to reduce rumination and its maladaptive consequences.	

During the development process, an initial feasibility study was conducted to consult with prospective end users. Participatory workshops were held to obtain feedback on the usability and acceptability of the app content and to discuss preliminary plans for the app structure, visual layout, and features [69]. Before these workshops, participants, selected based on their rumination levels, were asked to engage with a web-based version of the proposed app content (hosted via Qualtrics; SAP SE) for 4 weeks. Participants were asked to complete a series of pre- and posttests online, designed to mimic the randomized controlled trial protocol for this trial (see the “Methods” section, except for the BEAQ measure). A series of attentional tests was also included in the online assessment to control for response reliability. To be included in the analysis, participants were required to complete the first 2 weeks of content in full, followed by 5 of the 6 exercises from weeks 3 and 4.

Of the initial 687 participants who registered interest in participating, a sample of 55 was selected through prescreening using the PTQ. Participants were selected if their overall score was 1 SD above average. Of these, 21 participants completed all measures and were included in the quantitative analysis. Four participants volunteered to engage in participatory workshops and took part in semistructured interviews with members of the research team. The semistructured interviews focused on (1) factors determining compliance and strategies to overcome challenges in following the 4-week program; (2) strong and weak aspects identified in the program content; (3) accessibility of psychoeducational content in terms of language, clarity, and length; (4) strong and weak aspects of the user experience; (5) evaluation of different exercise types; and (6) evaluation of a mock-up for apps, with users having previous experience following the 4-week program.

Overall, data from the developmental feasibility study suggested that engagement with app content resulted in reductions in EA (comparison of pre- and postintervention measures; *P*<.001) and perseverative thinking (*P*<.001), including across all 3 subscales: mental load (*P*=.001), unproductiveness (*P*=.003), and core features (*P*=.002). While cognitive avoidance did not reduce significantly overall (*P*=.07), the descriptive reduction suggested an impact of the psychoeducational program on awareness. Thought substitution was significantly elevated after participation (*P*=.06), which may indicate increased awareness of this mechanism.

Because of the small sample size for participatory workshops, the data were not subjected to a formal analysis protocol and are unlikely to have reached saturation at this stage. However, feedback indicated that users found the intervention content and exercises interesting, with some participants reporting that they would have preferred more content to engage with. Additionally, participants requested that the app include an option to make personal notes invisible to the research team, provide links to additional information relevant to rumination and avoidance, and allow for text narration and audio recordings as alternatives to text entry.

Following user feedback, the app was updated to include a link providing further information about the research and the research team, along with relevant content details and additional external resources. Future versions of the app aim to incorporate further personalization options, such as voice narration, audio recording features, and a private notes function.

#### Remote Acquisition/Storage of Assessment Data

Data obtained through daily sampling are securely stored remotely via Firebase and are accessible only to authorized members of the research team, in compliance with General Data Protection Regulation (GDPR) requirements.

To capture user experiences during testing, the app also uses the CORTO method [82]. These data are likewise securely stored via Firebase and are accessible only to members of the research team.

### Statistical Analysis

#### Data Exclusion

Compliance will be defined as the completion of both the first and second weeks of the intervention content in full, along with at least 5 out of 6 exercises from weeks 3 and 4. For EMA, a compliance threshold of 80% will be used, consistent with previous research using EMIs in adult populations [90]. Additionally, the study will control for time spent within the app and the length of responses to open-ended questions.

In addition to the aforementioned compliance measures, participants who fail both attentional checks embedded within the assessments will be excluded from analyses.

#### Analysis of Trial Outcome Data

##### Primary Outcomes

Changes in outcomes across pre-, post-, and follow-up assessments (PTQ, EBQ, PBRS, NBRS, HADS, CAQ, and BEAQ) will be analyzed using mixed-design ANOVA across all 4 conditions. Additionally, multilevel models—treating daily observations as level 1 and participants as level 2—will be used to assess associations between avoidance and mood, as well as between rumination and mood, and to examine whether these associations are moderated by condition.

##### Secondary Outcomes

Mixed-design analysis of covariance will be conducted within the 2 intervention groups to examine whether time spent engaging with the app content serves as a covariate influencing intervention outcomes.

In-app user feedback collected through the CORTO method will be deductively coded following the process outlined by Saldana [91], using the 4 overarching categories proposed by Lukka et al [82]—(1) contextual use, (2) interaction-elicited emotional experience, (3) usability, and (4) technical issues—as a guiding framework. More detailed subdomains will be explored within each category.

### Ethics Approval

Ethical approval for the trial was obtained from the Ethics Committee of SWPS University, Katowice Faculty (ethical approval number WKEB90/11/2023).

## Results

Recruitment and enrollment for the trial are expected to begin in May 2025, with completion anticipated by July 2025. Data collection will conclude once the target sample size for each of the 4 conditions has been met. The main results of the trial are expected to be submitted for publication by October 2025. In addition to a formal publication in a peer-reviewed journal, the trial outcomes are anticipated to be presented in a summarized form at a relevant international conference.

## Discussion

### Study Strengths and Challenges

This paper outlines the protocol for a randomized controlled trial of an mHealth EMI aimed at reducing EA in the context of rumination. The primary objective of the trial is to investigate whether a reduction in EA through EMI intervention can lead to a decrease in RNT and its maladaptive consequences. Additionally, by implementing the CORTO method, the trial will gather user data on app usability and acceptability, identifying areas for continuous improvement and development.

While the primary objective of this study is to examine the role of EA, we are also collecting data on the use of the app itself. This information will inform its further development and potential efficacy testing in various settings. The app, as it currently stands, and its intervention content have been developed based on our formative user-experience research. While this approach is expected to enhance acceptability, usability, and user retention, and despite the app being thoroughly tested for issues, potential challenges during deployment to real-world users cannot be ruled out. For example, the app currently does not time-lock intervention modules and instead allows users to progress to the next week’s content once the preceding week’s material has been successfully completed. While this approach provides users with flexibility in how they complete the intervention, as recommended by prior studies [92,93], there is a possibility that some users may progress through the content more quickly than intended. This could potentially lead to poor information retention and a diminished impact of the intervention.

Definitions and reporting of compliance can vary significantly across studies of digital interventions [46]. The design of our app enables the remote collection of both daily assessment data and app usage metrics, allowing for precise measurement of engagement and, consequently, intervention compliance. For this study, we have adopted a compliance rate of 80%, consistent with a previous study using EMI and daily assessments with adult populations [89].

The incorporation of CORTO implementation enables the collection of real-time feedback from users regarding app usability and acceptability, addressing some of the limitations of retrospective interviews [82]. This approach acknowledges the central role of users as active participants in the treatment program and enhances the specificity of the feedback obtained [94]. As CORTO depends on sustained engagement with the digital tool under review, authors recommend considering survivorship bias when interpreting the data [82].

To address some of the limitations associated with using retrospective measures alone, the effectiveness of the intervention will also be assessed by analyzing daily sampling measures, as recommended by Heron and Smyth [43]. Additionally, we plan to compare the standalone version of the intervention with one that includes asynchronous support from CBT professionals. A recent meta-review of meta-analyses [95] suggested that guided interventions may be more effective and improve compliance; however, it notes that the quality of evidence is often low and that not all meta-analyses systematically compare the impact of guidance. Therefore, it is important to explore the effect of guidance. While including professional support can significantly enhance the intervention’s effectiveness, it also increases the cost of large-scale implementation and limits its potential for widespread dissemination. For this reason, a compromise solution involving asynchronous support was chosen.

The outcomes of the trial will be reported in a peer-reviewed publication following the mHealth Evidence Reporting and Assessment guidelines, as outlined by the World Health Organization mHealth Technical Evidence Review Group [96]. These guidelines are designed to enhance the quality and clarity of mHealth evaluation reporting by specifying where detailed information should be provided regarding intervention content, implementation context, and relevant technical aspects.

### Conclusions

It is anticipated that the outcomes of this trial will not only provide insights into the clinical effectiveness of the app but also will primarily offer valuable theoretical understanding of the role of EA in maladaptive rumination. Additionally, the user feedback collected through the CORTO implementation will contribute to improving the app’s usability and acceptability in future iterations and further enrich the growing body of literature in this field.

## Appendix

Multimedia Appendix 1 Daily assessment items.

Multimedia Appendix 2 In-app CORTO (Contextual, One-Item, Repeated, Timely, Open-Ended) items.

## Abbreviations

BEAQ: Brief Experiential Avoidance Questionnaire
CAQ: Cognitive Avoidance Questionnaire
CBT: cognitive behavioral therapy
CORTO: Contextual, One-Item, Repeated, Timely, Open-Ended
EA: experiential avoidance
EBQ: Emotions Beliefs Questionnaire
eHEALS: eHealth Literacy Scale
EMA: ecological momentary assessment
EMI: ecological momentary intervention
GDPR: General Data Protection Regulation
H_Ex_A_Go_N: habit, executive function impairment, abstract processing mode, goal discrepancies triggering rumination, and the negative valence of rumination content
HADS: Hospital Anxiety and Depression Scale
mHealth: mobile health
NBRS: Negative Beliefs About Rumination Scale
PBRS: Positive Beliefs About Rumination Scale
PTQ: Perseverative Thinking Questionnaire
RFCBT: rumination-focused cognitive behavioral therapy
RNT: repetitive negative thinking

## Data Availability

Once the outcomes of the trial have been published, data will be made publicly available via the Open Science Framework.
