# Supplementary material for: A Mobile Ecological Momentary Intervention for Reducing Experiential Avoidance in the Context of Rumination: Protocol for a Randomized Controlled Trial
Source: JMIR Res Protoc. 2025 May 27;14:e66067. doi: 10.2196/66067 (PMC12152439; doi:10.2196/66067)
Supplement: Multimedia Appendix 1 [file resprot_v14i1e66067_app1.docx]

Appendix 1: Daily assessment items.

| Variable (dimension) | Original item (scale) |
| --- | --- |
| RNT |  |
| (Feelings) | At the moment I am thinking about my feelings  (1: “not at all”, 7: “very much”) |
| (Problems) | At the moment I am thinking about my problems  (1: “not at all”, 7: “very much”) |
| (Past) | At the moment I am thinking about upsetting memories  (1: “not at all”, 7: “very much”) |
| (Future) | At the moment I am thinking about negative future situations  (1: “not at all”, 7: “very much”) |
| (Duration) | How long have you been thinking about these topics up to this moment?  (1: “not at all”, 7: “more than 120 min.”) |
| (Distress) | How much do you feel weighed down by these thoughts at this moment?  (1: “not at all”, 7: “very much”) |
| (Repetitiveness) | The same thoughts keep going through my mind again and again. (1: “not at all”, 7: “very much”) |
| (Intrusiveness) | Thoughts come to my mind without me wanting them to.  (1: “not at all”, 7: “very much”) |
| (Uncontrollability) | I get stuck on certain issues and can’t move on.  (1: “not at all”, 7: “very much”) |
| Depression | How was your day today? (1: “very unsuccessful”, 7: “very successful”) |
|  | To what extent did you think positively about yourself today?  (1: “not at all”, 7: “very much”) |
|  | How do you feel about tomorrow?  (1: “very negative”, 7: “very positive”) |
| Affect | How did you feel today? |
|  | Nervous/anxious  (1: “not at all”, 7: “very much”) |
|  | Sad  (1: “not at all”, 7: “very much”) |
|  | Angry  (1: “not at all”, 7: “very much”) |
|  | Disgusted  (1: “not at all”, 7: “very much”) |
| *Open-question (following previous item)* | Choose the emotion that you felt the strongest - what useful function could it have played for you today?  *[open question]* |
| Goals | Choose one goal that was important to you today.  *[open question]* |
|  | How important was this goal?  (1: “not at all”, 7: “very much”) |
|  | To what extent did you manage to achieve this goal?  (1: “not at all”, 7: “very much”) |
| Rumination outcomes |  |
| Productiveness | How productive was your rumination today?  (1: “not at all”, 7: “very much”) |
| Emotional discomfort | How difficult/painful/comfortable was this reflection for you?  (1: “not at all”, 7: “very much”) |
| Motivation | Did it motivate you to take specific action?  (1: “definitely not, 7: “definitely yes”) |
