# Supplementary material for: A Mobile Ecological Momentary Intervention for Reducing Experiential Avoidance in the Context of Rumination: Protocol for a Randomized Controlled Trial
Source: JMIR Res Protoc. 2025 May 27;14:e66067. doi: 10.2196/66067 (PMC12152439; doi:10.2196/66067)
Supplement: Multimedia Appendix 2 [file resprot_v14i1e66067_app2.docx]

Appendix 2: In-app CORTO items

| Do you like using the application?  (1: “not at all”, 7: “very much”) |
| --- |
| How would you rate the difficulty level of using the application?  (1: “definitely too easy”, 7: “definitely too difficult”) |
| Would you like to share any thoughts about the app?  *(open-ended question)* |
